# Actin Turnover Is Required for Myosin-Dependent Mitochondrial Movements in *Arabidopsis* Root Hairs

**DOI:** 10.1371/journal.pone.0005961

**Published:** 2009-06-18

**Authors:** Maozhong Zheng, Martina Beck, Jens Müller, Tong Chen, Xiaohua Wang, Feng Wang, Qinli Wang, Yuqing Wang, František Baluška, David C. Logan, Jozef Šamaj, Jinxing Lin

**Affiliations:** 1 Key Laboratory of Photosynthesis and Molecular Environmental Physiology, Institute of Botany, Chinese Academy of Sciences, Beijing, China; 2 Graduate School of Chinese Academy of Sciences, Beijing, China; 3 Institute of Cellular and Molecular Botany, Rheinische Friedrich-Wilhelms-University Bonn, Department of Plant Cell Biology, Bonn, Germany; 4 Institute of Botany, Slovak Academy of Sciences, Bratislava, Slovak Republic; 5 Department of Biology, University of Saskatchewan, Saskatoon, Saskatchewan, Canada; 6 Institute of Plant Genetics and Biotechnology, Slovak Academy of Sciences, Nitra, Slovak Republic; 7 Faculty of Science, Palacký University Olomouc, Olomouc, Czech Republic; Purdue University, United States of America

## Abstract

**Background:**

Previous studies have shown that plant mitochondrial movements are myosin-based along actin filaments, which undergo continuous turnover by the exchange of actin subunits from existing filaments. Although earlier studies revealed that actin filament dynamics are essential for many functions of the actin cytoskeleton, there are little data connecting actin dynamics and mitochondrial movements.

**Methodology/Principal Findings:**

We addressed the role of actin filament dynamics in the control of mitochondrial movements by treating cells with various pharmaceuticals that affect actin filament assembly and disassembly. Confocal microscopy of *Arabidopsis thaliana* root hairs expressing GFP-FABD2 as an actin filament reporter showed that mitochondrial distribution was in agreement with the arrangement of actin filaments in root hairs at different developmental stages. Analyses of mitochondrial trajectories and instantaneous velocities immediately following pharmacological perturbation of the cytoskeleton using variable-angle evanescent wave microscopy and/or spinning disk confocal microscopy revealed that mitochondrial velocities were regulated by myosin activity and actin filament dynamics. Furthermore, simultaneous visualization of mitochondria and actin filaments suggested that mitochondrial positioning might involve depolymerization of actin filaments on the surface of mitochondria.

**Conclusions/Significance:**

Base on these results we propose a mechanism for the regulation of mitochondrial speed of movements, positioning, and direction of movements that combines the coordinated activity of myosin and the rate of actin turnover, together with microtubule dynamics, which directs the positioning of actin polymerization events.

## Introduction

Mitochondria serve as energy-producing organelles in plants and other eukaryotes, and they are known to associate with energy-consuming organelles and structures, such as the nucleus, rough endoplasmic reticulum, and plasma membrane [1]. In addition, recent research has demonstrated that mitochondria serve as fundamental elements in intracellular signaling. For example, mitochondria play a role in modulating the spatiotemporal features of a [Ca^2+^]_c_ signal [2]–[4] and are crucial participants in cell death programs [5]–[7]. Previous studies using mammalian cells have suggested that strategic mitochondrial localization at particular subcellular sites was required both for the provision of energy and for intracellular signaling [8]. Because of the importance of mitochondrial distribution for cellular activities, the mechanism of mitochondrial movements has received considerable attention, particularly in yeast and animal cells.

Active mitochondrial movements involve a close interaction with the cytoskeleton, in particular, with actin filaments or with microtubules. A number of studies have shown that both microfilaments and microtubules function in mitochondrial movements, although the contribution of each cytoskeletal element varied considerably depending on specific cell types and organisms. In most animal cells and also some algae and protists, mitochondrial movement is mainly dependent on microtubules [4], [9]–[11], and mediated by molecular motor proteins such as kinesins and dyneins that move the cargo toward the plus-end or minus-end of microtubules, respectively [12]. However, studies in neuronal axons [13], [14] and in insect cells [15] revealed that actin filaments could also serve as tracks for mitochondrial transport. In higher plants it has been shown that mitochondria are closely associated with microfilaments and mitochondrial translocation is mainly dependent on intact microfilaments, in contrast to studies using yeast or animal cells [16]–[19].

F-actin undergoes turnover: the exchange of actin subunits from existing filaments through polymerization at the barbed ends of filaments in conjunction with depolymerization at the pointed ends enabling increases and decreases in the length of polymerized actin filaments [20]. Apart from actin turnover, more complex dynamics of actin filaments have been recognized, including the flow of polymerized actin filaments, elongation and shortening of polymerized actin filaments, and other morphological changes [21], [22]. Specific functions of the actin cytoskeleton depend on how actin filaments are distributed and arranged, and how their behavior is modified by proteins that directly associate with both intact polymers and their monomer subunit [23]–[25]. Cytoskeletal dynamics play an integral role in cellular metamorphosis, organelle trafficking and endocytosis. For example, a polarized actin polymerization process is involved in the movement of bacteria inside the host cytosol [26]. Furthermore, it has been demonstrated that actin cables serve as “conveyor belts” to drive retrograde movement of mitochondria, endosomes, and actin patches in *Saccharomyces cerevisiae* [27], [28]. Recently, it has also been suggested that endosomal movements in *Medicago trunculata* root hairs are driven by actin polymerization [29]. To date, no research on the function of actin filament dynamics in driving mitochondrial movements has been reported in tip-growing cells.

To study the roles of the cytoskeleton in many aspects of plant growth, several cytoskeletal inhibitors, such as latrunculin B (LatB), cytochalasin D (CD), jasplakinolide (Jas), oryzalin, taxol and 2,3-butanedione 2-monoxime (BDM) have been widely used [30]. Using pharmacological inhibitors, Van Gestel et al. reported that plant mitochondria moved on F-actin, but that their positioning in the cortical cytoplasm depends on F-actin and microtubules [17]. Sheahan et al. found that inhibiting myosin activity and depolymerizing F-actin stopped vectorial mitochondrial movement, whereas disrupting microtubules slightly reduced mitochondrial movement [18]. Recently, Doniwa et al. demonstrated that mitochondria moved along actin filaments in BY-2 cells and *Arabidopsis* epidermal cells [19]. However, the inhibitors were used at high concentrations and applied for long time frames in these experiments, which makes deciphering of the role of actin filament dynamics in plant mitochondrial movements impossible and may cause nonspecific effects of drug treatments.

Root hairs show strictly polar cell-expansion called tip growth. Within the growing root hair the cytoplasm itself exhibits a high degree of polarized zonation. Growing hairs have a tip region free of detectable bundles of actin filaments, behind which is a subapical region of net axially-aligned fine F-actin, followed by a basal region showing thicker bundles of actin filaments [31]. Correspondingly, it has been reported that a tip-focused G-actin gradient is present in growing root hairs that is not present in fully-grown root hairs [32]. Therefore, growing root hairs provide an excellent model system to study actin filament dynamics in relation to mitochondrial movements.

In this investigation we co-visualized actin filaments and mitochondria in living growing root hairs using stably transformed *Arabidopsis thaliana* seedlings expressing GFP-FABD2 [33] co-labelled with vital dye MitoTracker Red CMXRos. Additionally, the trajectories and instantaneous velocities of mitochondria were analyzed immediately following pharmacological perturbation of the cytoskeleton using variable-angle evanescent wave microscopy (EWM) and/or spinning disc confocal microscopy (SDCM) in wild type *Arabidopsis*, or in an *Arabidopsis* line stably transformed with a mito-GFP construct. Finally, cross-correlation analysis was used to compare mitochondrial movements with the status of actin filaments in order to decipher the relationship between mitochondria movements and the actin cytoskeleton.

## Results

### Mitochondrial distribution and actin filament organization in root hairs at different developmental stages

**Figure 1:**
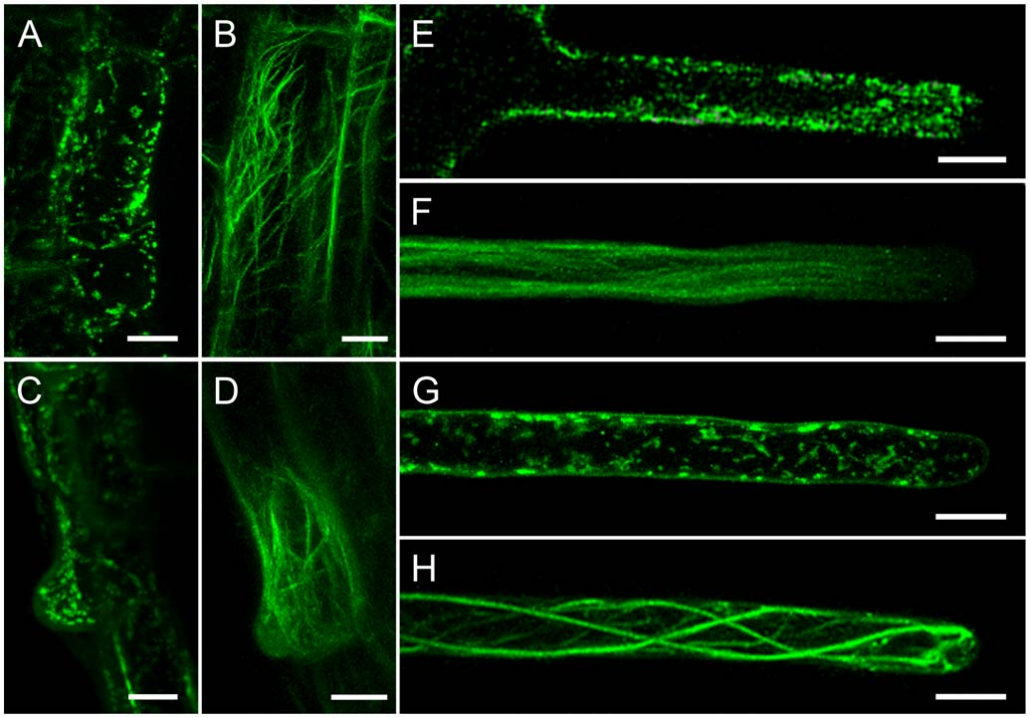
Mitochondrial distribution and actin organization in root hairs at different developmental stages. Mitochondria are labeled with MitoTracker, actin is visualized using *GFP-FABD2*. The single pictures represent a stack of all optical sections through these cells visualized by spinning disc confocal microscopy. A. Mitochondria were evenly distributed in the cortical cytoplasm in pre-bulge of trichoblast. Scale bar = 10 µm. B. Thick actin filaments formed a network in prebulge trichoblast. Scale bar = 10 µm. C. Mitochondrial distribution in a bulge. Note that mitochondria were agreggated within the region where the bulge formed. Scale bar = 10 µm. D. Actin filaments are undetected in the tip of bulge, whereas fine actin filaments are visible in the site of bulge outgrowth. Scale bar = 10 µm. E. Mitochondrial distribution in a fast elongating root hair. Note the large number of mitochondria in the subapical region. Scale bar = 10 µm. F. Actin filaments in fast elongating root hairs. Note the large number of fine actin filaments in the subacpcial region and absence of actin filaments in the apical region. Scale bar = 10 µm. G. In the full growth root hair, mitochondrial density significantly decreased and showed uniform distribution. Scale bar = 10 µm. H. Thick actin filaments arranged in the full growth root hair. Scale bar = 10 µm

Mitochondria in trichoblasts at the pre-bulge stage were evenly distributed in the cortical cytoplasm (Fig. 1A) containing actin bundles with an average thickness of 0.72±0.12 µm (n = 15 cells) (Fig. 1B). When trichoblast cells formed bulges, mitochondria aggregated within the bulge (Fig. 1C), the average thickness of actin bundles were reduced to 0.41±0.10 µm (n = 14 cells), and fine actin filaments were present at the site of bulge outgrowth (Fig. 1D). In elongating root hairs, mitochondrial density increased markedly while mitochondrial distribution showed a subapical to base gradient (Fig. 1E). Visualization of actin filaments in fast elongating root hairs expressing GFP-FABD2 revealed that extensive arrays of thick actin bundles at the base extended towards tip, dispersed into fine filaments in the subapical region and became much finer in the apex, eventually becoming undetectable (Fig. 1F). When G-actin polymerized into thick stable actin filaments in terminally differentiated root hairs that had stopped growing (Fig. 1H), mitochondria were uniformly distributed in the cytoplasm and their density declined to 35.7% of that in elongating root hairs (Fig. 1G).

### Characterization of the movement of individual mitochondria by EWM

**Figure 2:**
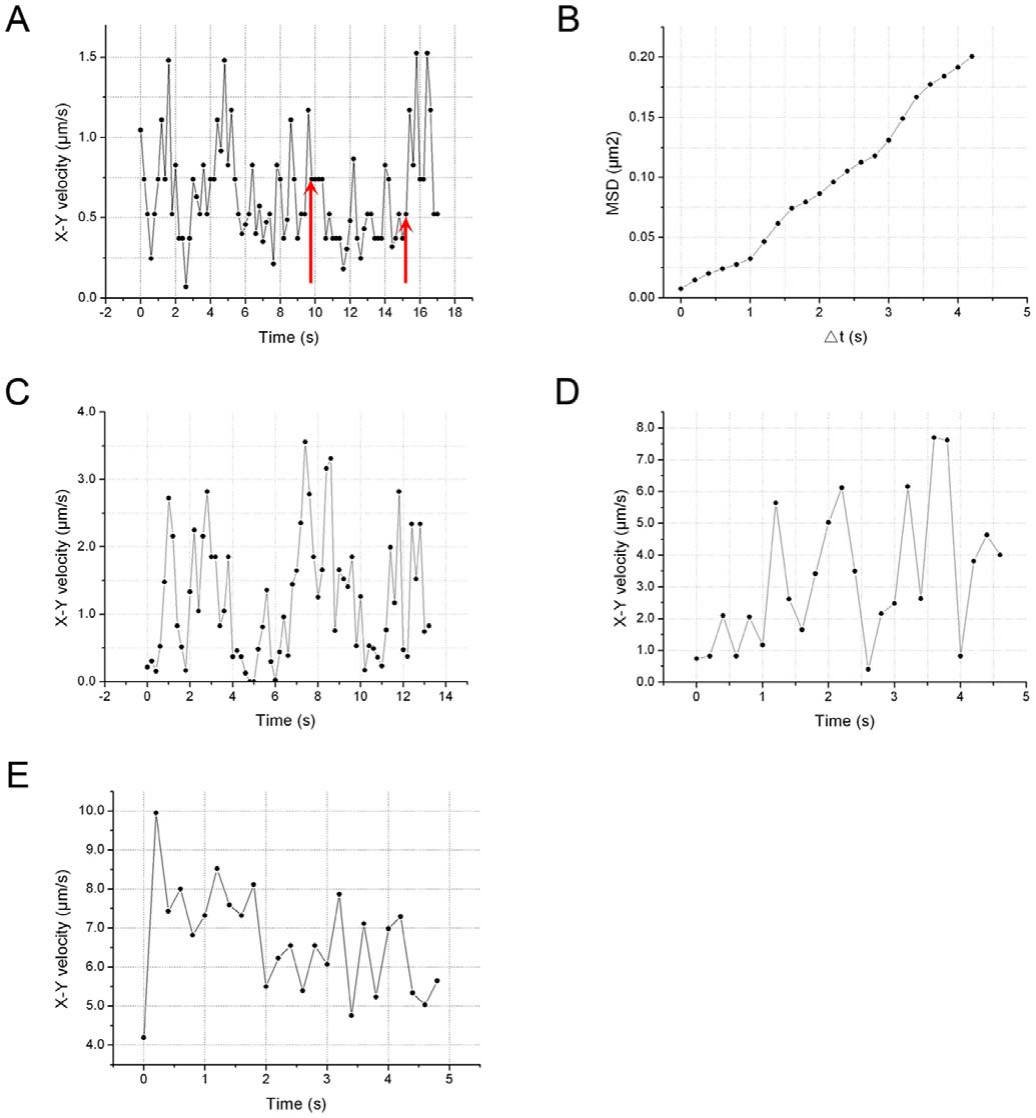
Mitochondrial linear movements in *Arabidopsis* root hairs. Mitochondria in wild-type *Arabidopsis* are labeled with MitoTracker (red) and their movements in a single focal plane were recorded by evanescent wave microscope at 200 millisecond intervals. A. Plot of the x-y velocity of a mitochondrion moving in the region 5–30 µm from the tip. Note that the instantaneous velocities ranged from 0.07 µm/sec to 1.53 µm/sec. From 9.8 s to 15.2 s as indicated by arrows, the mitochondrion behaved rotations and oscillations at low instantaneous velocities. B. The mean square displacement (MSD) of the pause (highlighted the start and the end by arrows in A) plotted against time interval Δt. C. Plot of the x-y velocity of a mitochondrion moving in the region 30–80 µm from the tip. Note that the instantaneous velocities ranged from 0.06 µm/sec to 3.56 µm/sec. D. Plot of the x-y velocity of a mitochondrion moving in the basal shank. Note that the instantaneous velocities ranged from 0.41 µm/sec to 7.70 µm/sec. E. Plot of the x-y velocity of a mitochondrion moving in fully grown root hairs. Note that the instantaneous velocities ranged from 4.19 µm/sec to 9.96 µm/sec.

Previously, we used EWM to study endosomal movements in growing pollen tubes [34]. In the current study, mitochondria visualized either with mito-GFP [35], or with MitoTracker, showed active movements in epidermal root cells and in root hairs. Using the time-lapse images obtained by EWM with 200 milliseconds intervals in a single focal plane, we analyzed the x-y plane velocities and trajectories of individual mitochondria. The results demonstrate that mitochondria can undergo a wide range of rates and directions of linear movement (as shown in video S1). Furthermore, measurement and statistical analysis revealed that a gradient of mitochondrial velocity occurred along the long axis of growing root hairs (Table 1). In the region 5–30 µm from the apex, mitochondria moved at an average velocity of 0.64±0.32 µm/sec (n = 15 root hairs, 985 mitochondria), with a maximum velocity of 2.63 µm/sec. As the distance from the apex increased to 30–80 µm, the average velocity of mitochondria increased to 1.80±1.03 µm/sec (n = 14 root hairs, 990 mitochondria), with a maximum velocity of 5.97 µm/sec. In the base of growing root hairs, the average velocity of mitochondria increased further to 3.45±2.13 µm/sec (n = 12 root hairs, 893 mitochondria), with a maximum velocity of 9.96 µm/sec. However, this velocity gradient was not apparent in fully grown root hairs, in which mitochondria moved throughout the entire length of the root hair at an average velocity of 4.03±2.06 µm/sec (n = 13 root hairs, 806 mitochondria), with a maximum velocity of 10.2 µm/sec (Fig. 2E).

**Table 1:** Overview of mitochondrial linear movements along the direction of growth in growing root hairs (n = 22 root hairs, 979 mitochondria).

Velocity of mitochondrial movement (µm/sec)	5–30 µm from apex	30–80 µm from apex	Base of root hair
Mean±SD	0.64±0.33	1.80±1.08	3.45±2.13
Maximum	2.63	5.97	9.96
Minimum	0.0054	0.021	0.41

To further characterize mitochondrial movement, the motility of individual mitochondria over time was plotted in an x-y velocity graph. Pauses were operationally defined as status that mitochondria lost their linear movement and instead exhibited complex behaviors, e.g., rotations, oscillations, and immobility. At the tip of the root hairs, mitochondria frequently moved at a lower velocity and paused more frequently than in the regions closer to the base. During pauses, mitochondrial instantaneous velocities declined to 0.5 µm/sec over 1 second. The velocities of an individual mitochondrion in the apical zone over a 17 sec period are presented in Fig. 2A. To characterize mitochondrial behavior during periods of pause, the mean squared displacement (MSD) at different time intervals during a pause (indicated by the red arrow) was calculated and these values were averaged to generate the MSD-versus-time plot (Fig. 2B). The diffusion coefficient in two dimensions, D^2^, was derived from the slope of a plot of the MSD-versus-time for interval Δt→0. The diffusion coefficient ranged from 1.75×10^−2^ µm^2^/sec to 9.1×10^−2^ µm^2^/sec, with an average value of 4.6×10^−2^±2.1×10^−2^ µm^2^/sec (n = 103 mitochondria), suggesting that mitochondrial motion was neither random nor Brownian during their pauses according to Stokes' law. In the region 30–80 µm from the tip and in the basal part of the growing root hairs, mitochondria moved more rapidly and paused less often, as indicated by a lower frequency of instantaneous velocities of less than 0.5 µm/sec (Fig. 2C and 2D).

### Effects of actin cytoskeleton disruption on mitochondrial movements

**Figure 3:**
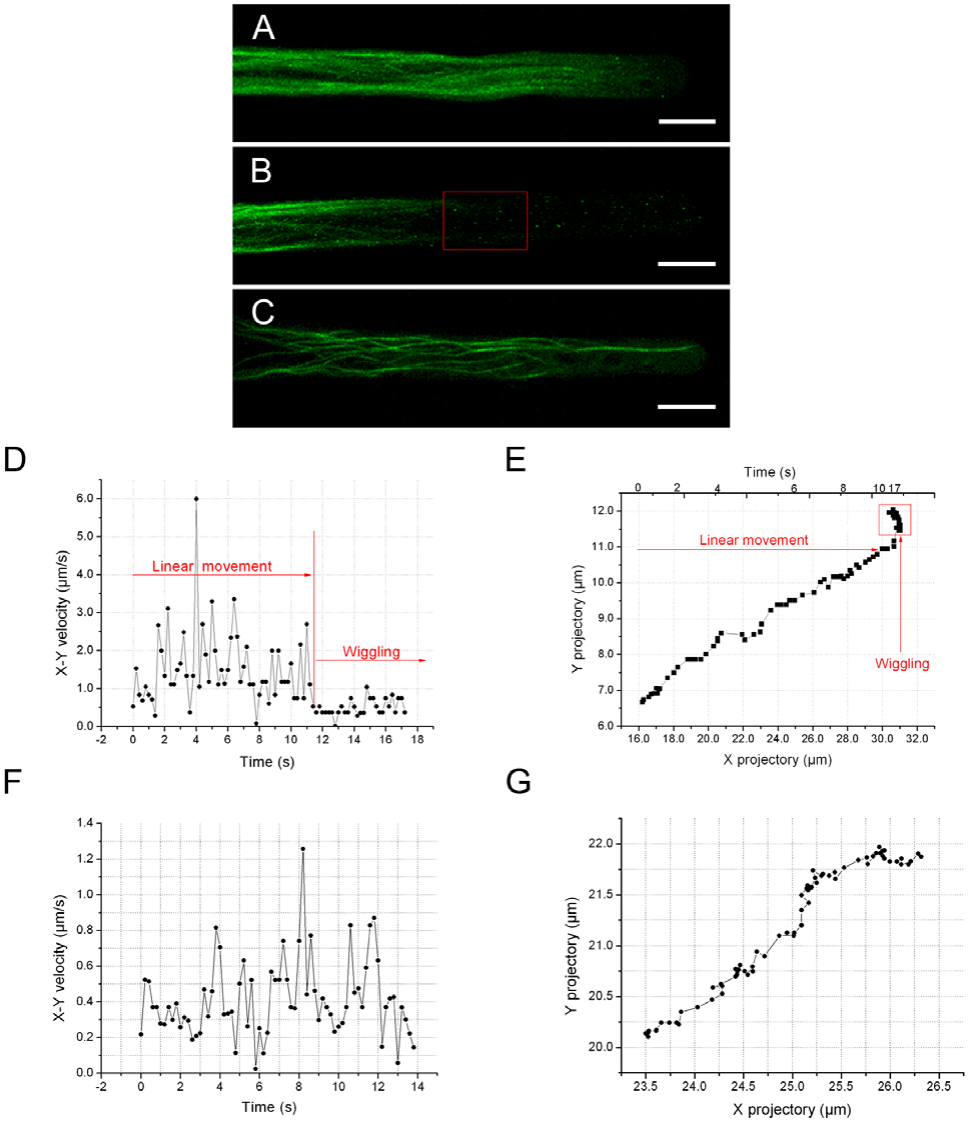
Effects of cytochalasin D (CD) and latrunculin B (LatB) on actin filaments and mitochondrial movements. A. Fluorescence micrograph showing actin filaments labeled with GFP-FABD2 in an untreated growing root hair of *Arabidopsis*. Note the fine actin filaments in the subapical region. Scale bar = 10 µm. B. Fluorescence micrograph showing actin filaments labeled with GFP-FABD2 in a growing root hair treated with 5 µM CD. Note that fine actin filaments were completely eliminated in the apical and subapical region. Scale bar = 10 µm. C. Fluorescence micrograph showing actin filaments in a growing root hair treated with 10 nM LatB. Note that thick actin filaments remained and the fine actin filaments disappeared. Scale bar = 10 µm. D. Plot of the x-y velocity of a mitochondrion moving from base to the tip in the region outlined by the square in B. Note the arrest of mitochondrial movement. E. Plot of x-y coordinates showing the same mitochondrion as in D wiggling or oscillating after the cessation of linear movement. F. Plot of the x-y velocity of a mitochondrion moving in the region 30–80 µm from the tip of a growing root hair treated with 10 nM LatB for 10 minutes. G. Plot of x-y coordinates showing the same mitochondrion as in F undergoing linear movement.

Treatment of seedlings with 5 µM CD for 10 minutes did not inhibit cytoplasmic streaming in any of the growing root hairs tested. Confocal microscopy demonstrated that use of 5 µM CD resulted, in the subapical area, in a decrease in GFP-FABD2 labeled actin structure, including the apparent disappearance of fine actin filaments and the appearance of very fine spots, whereas toward the base of the root hair the thick bundles of actin filaments remained intact (Fig. 3A and 3B). The effects of CD on mitochondrial movements showed typical dose- and time-dependent patterns. A notable effect of treatment with 5 µM CD for 10 minutes on mitochondrial movements was that linear movements were gradually replaced with wiggling motion or immobility, particularly in the apical region of growing root hairs. As shown in Fig. 3B and 3D, sudden inhibition of linear mitochondrial movement occurred when the mitochondrion arrived at a position 38.4 µm from the tip. Before this arrest, the mitochondria continued to move as fast as they did before drug application (Fig. 3E). As the time after treatment increased, more mitochondria lost their linear movement, until all mitochondria were either wiggling or immobile.

When treated with 10 nM LatB, most mitochondria became immobile within 2 minutes. Residual mitochondrial linear movement displayed an average velocity of 0.41±0.21 µm/sec (Fig. 3F). Although the instantaneous velocities were lower than 0.5 µm/sec, the video and analysis of it demonstrate that mitochondria moved vectorially (Fig. 3G). The thick actin filaments in the area between the basal and subacpical regions remained present when treated with LatB for 10 minutes, whereas the fluorescence of FABD2 in the apical region trailed off (Fig. 3C).

**Figure 4:**
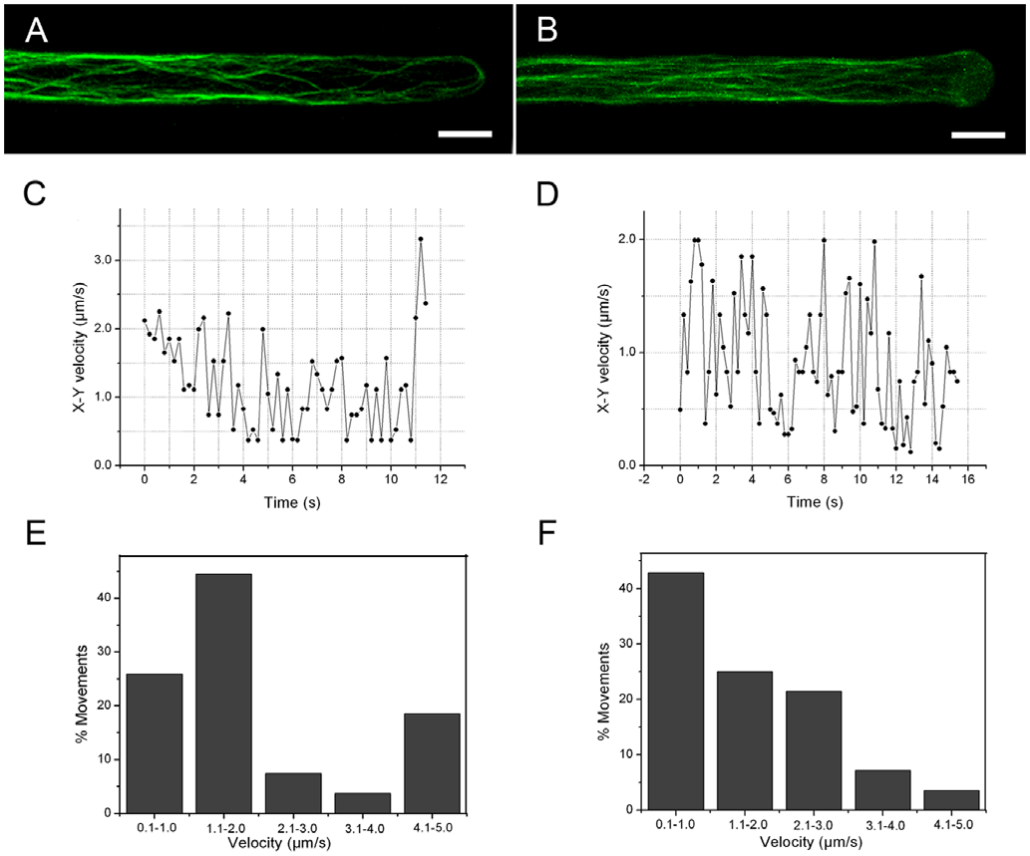
Effects of jasplakinolide (Jas) and 2, 3-butanedione 2-monoxime (BDM) on actin filaments and mitochondrial movements. A. A growing root hair treated with 100 nM Jas for 10 minutes. Note thick actin bundles aggregated at the margins and actin filaments looping into the apex. Scale bar = 10 µm. B. A growing root hair treated with 30 mM BDM for 10 minutes, showing fine actin filaments extending into the apex of the root hair and emergence of slightly thicker actin filaments. Scale bar = 10 µm. C. Plot of the x-y velocity of a mitochondrion moving in the region 30–80 µm from the tip of a growing root hair treated with Jas. D. Plot of the x-y velocity of a mitochondrion moving in a growing root hair treated with 30 mM BDM. E. Net rates and frequencies of mitochondrial movements in the region 0–40 µm from the tip of a growing root hair treated with Jas (n = 8 root hairs, 242 mittochondria). F. Net rates and frequencies of mitochondrial movements in the region 0–30 µm from the tip of growing root hairs treated with 10 mM BDM (n = 7 root hairs, 256 mitochondria).

Jasplakinolide (Jas), a cell-permeable macrocyclic peptide that inhibits F-actin turnover [36], [37], was used to investigate the role of F-actin turnover in mitochondrial movement. Treatment of GFP-FABD2 expressing seedlings with 100 nM Jas for 10–15 min resulted in an increase in GFP fluorescence adjacent to the plasma membrane: thick actin bundles aggregated at the cell margins and actin filaments looped into the root hair apex (Fig. 4A). At a distance of 30–80 µm from the tip, most mitochondria lost linear movement and were shown to wiggle into or out of the focal plane, deep in the interior cytoplasm. Other mitochondria that maintained linear movement in the cortical cytoplasm moved at velocities ranging from 3.25 to 0.25 µm/sec, with an average velocity of 1.04±0.52 µm/sec (n = 17 root hairs, 438 mitochondria), representing approximately 57.6% of the average velocity measured in control root hairs (Fig 4C). In contrast to the distribution and movement of mitochondria after treatment with CD, treatment with Jas resulted in mitochondria moving into the tip of the growing root hairs. An interesting phenomenon induced by Jas was that mitochondria moved at a range of velocities in the 0–30 µm region of the root hairs (Fig. 4E), with a maximal instantaneous velocity of 8.05 µm/sec.

### Myosin functions in mitochondrial movements

To test the dependency of mitochondrial movements on myosin activity, *Arabidopsis* seedlings were treated with the myosin inhibitor BDM. Different concentrations of BDM were tested to determine the optimal concentration for maximum inhibition of myosin while minimizing its potential side effects. When treated with 10 mM BDM for 10 minutes, no obvious change was detected in the organization of actin filaments in growing root hairs (data not shown). Mitochondria remained motile although their average velocity in the 30–80 µm region of growing root hairs was reduced to 1.27±0.69 µm/sec (n = 13 root hairs, 588 mitochondria), representing approximately 70% of the veolicty measured in the control. As observed following treatment with Jas (see above, Fig. 4E) mitochondrial movements in the 0–30 µm region were highly variable ranging from 0.57 µm/sec to 4.58 µm/sec (Fig. 4F).

When treated with the higher concentration of 30 mM BDM for 10 minutes, fine actin filaments extended into the apex of growing root hairs and became slightly thicker than in the control (Fig. 4B). The average velocity of all mitochondria declined to 0.83±0.19 µm/sec (n = 14 root hairs, 687 mitochondria), with instantaneous velocities ranging from 2.82 to 0.19 µm/sec throughout the whole root hair (Fig. 4D). Because an obvious side effect of the treatment in actin filament remodeling was observed (Figure S1) and the reduction in mitochondrial velocity was moderate (Figure S2) when the concentration of BDM higher than 40 mM was used, the 30 mM BDM was selected as the reasonable concentration to minimize the side effects. Finally, *Arabidopsis* seedlings were exposed to a combination of BDM and Jas to determine whether the residual linear mitochondrial movements seen after BDM treatment were driven by actin turnover. The combination of BDM (30 mM) and Jas (100 nM) led to complete cessation of mitochondrial movement and a dispersal of mitochondria throughout the cytoplasm.

### Effects of microtubule disruption on mitochondrial movements

**Figure 5:**
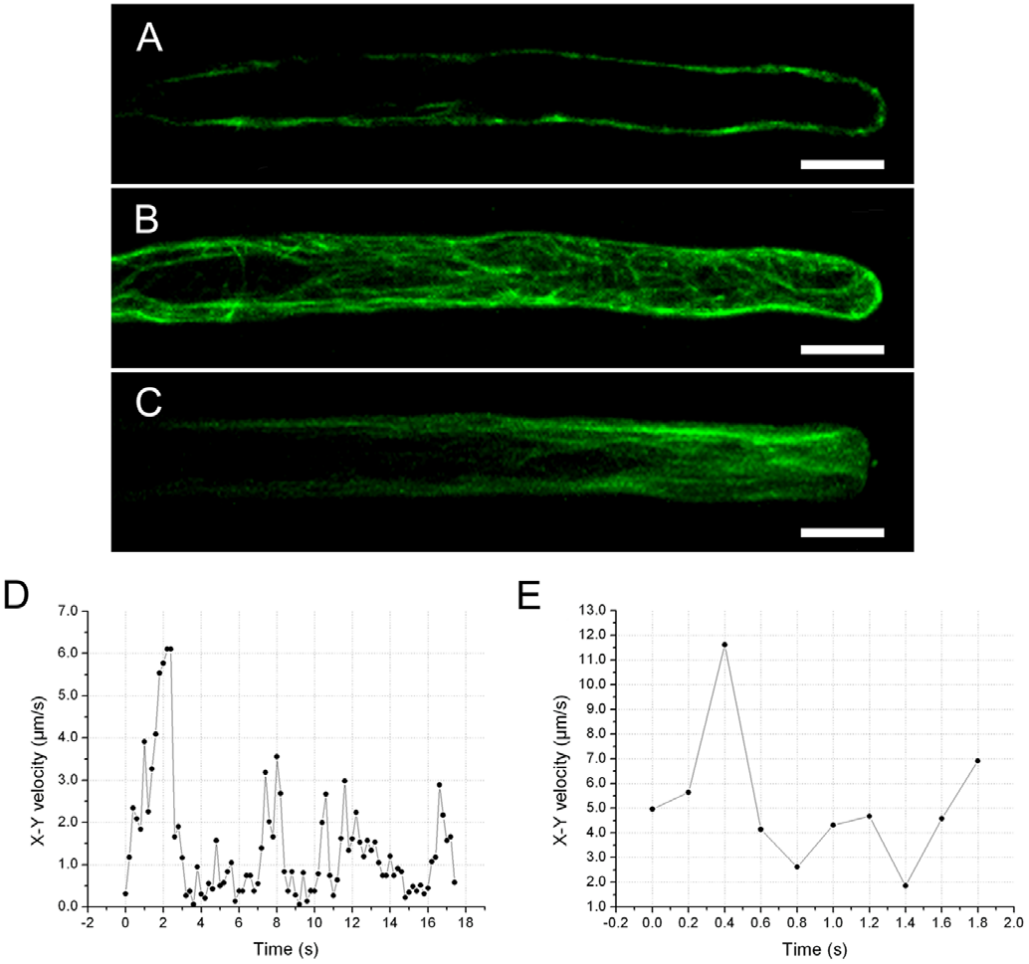
Effects of microtubule-active drugs on actin filaments and mitochondrial movements. A. A single optical section of interior cytoplasm of the root hair of B. Note that no actin filaments are visible in the interior cytoplasm. Scale bar = 10 µm. B. A growing root hair treated with 10 µM oryzalin displaying dispersed and curved actin bundles around the cortical cytoplasm and in the base of the hair. Scale bar = 10 µm. C. A growing root hair treated with 5 µM taxol showing an increase in fluorescence in the subapical and apical regions and an aggregation of thick actin bundles in the very tip of the hair, concomitant with a decrease in the basal shank. Scale bar = 10 µm. D. Plot of the x-y velocity of a mitochondrion moving in the region 0–80 µm from the tip of a growing root hair treated with 10 µM oryzalin. E. Plot of the x-y velocity of a mitochondrion moving in the region 0–80 µm from the tip of a growing root hair treated with 5 µM taxol.

To determine whether microtubules play a role in the regulation of mitochondrial movements, *Arabidopsis* seedlings were treated with oryzalin and taxol. Exposure of *Arabidopsis* seedlings to 10 µM oryzalin led to an almost complete depletion of actin filaments from the interior cytoplasm of the subapical region of growing root hairs (Fig 5A). Such root hairs displayed dispersed and curved actin bundles in the cortical cytoplasm (Fig. 5B). Mitochondria congregated in the cortical cytoplasm and their trajectories tended to curve. The polar gradient of mitochondrial velocity along the long axis of the root hair was no longer observed in both 0–30 µm and 30–80 µm regions upon treatment with oryzalin. When plotting velocity over time, it was apparent that mitochondria remained capable of high instantaneous velocities, and the maximum velocity was 6.1 µm/sec (Fig. 5D). Compared with mitochondria in the control root hairs which moved with a similar velocity (compare with Fig. 2C and 2D), oryzalin treatment caused mitochondria to pause more frequently (0.38 pauses per second) and for a longer time (0.79 seconds per pause) (Fig. 5D).

Unlike oryzalin, treatment with 5 µM taxol did not alter the cytoarchitecture of growing root hairs and did not affect the general distribution of the GFP-FABD2 actin reporter, however fluorescence increased in the 0–30 µm region showing the presence of thick actin bundles in the apex, and sharply decreased in the basal region (over 80 µm from the apex) (Fig 5C). Statistical analysis showed that the mitochondrial velocity gradient was no longer detectable in the 0–30 µm and 30–80 µm regions and that approximately 9.3% of the total mitochondrial population became immobile. However, the majority of mitochondria moved faster with fewer pauses: the average velocity was 3.9±1.91 µm/sec (n = 11 root hairs, 671 mitochondria), with a maximum instantaneous velocity of 11.6 µm/sec, representing an increase of 16.6% if compared with that found in the control (Fig. 5E).

### Simultaneous visualization of mitochondrial movements and dynamics of the actin cytoskeleton

**Figure 6:**
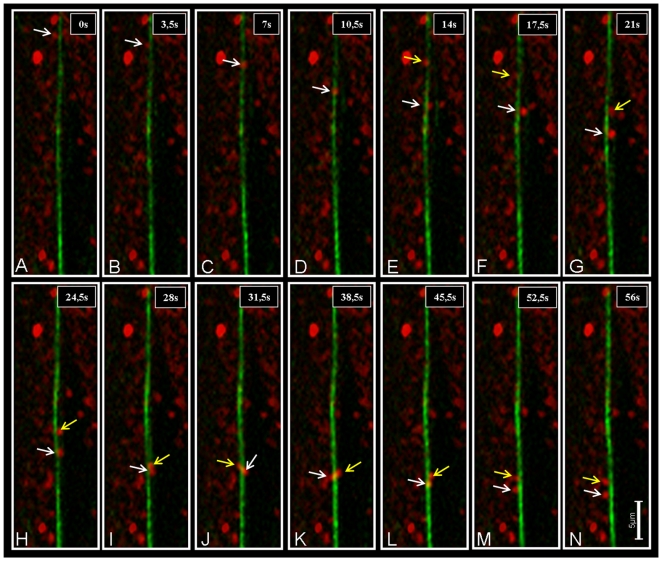
Movement of two mitochondria along an actin filament cable. In images A–G a mitochondrion (white arrow) is turning around the axis of the filament while moving downwards. When the second mitochondrion (yellow arrow) appears, it contacts the first (picture I). In contrast to the first turn of the single mitochondrion, there is no downward movement of the touching mitochondria while they are turning around the axis of the actin bundle. Mitochondria are labeled with MitoTracker (red), actin is visualized using *GFP-FABD2* (green). The single pictures represent a single frame of a “flat merged” projection (each comprised of 14 images in z-axis) of a 4–D time series. The 5 µm scale bar in N applies for all images in this figure.

When mitochondria and actin filaments in the root hairs of *Arabidopsis* seedlings were simultaneously visualized using MitoTracker and GFP-FABD2 respectively, fluorescence microscopy revealed that mitochondria were closely aligned with actin filaments. Additionally, mitochondrial movements followed trajectories defined by actin filaments (video S2). During observation, mitochondria were mostly undergoing vectorial transport, a wiggling motion, or they were immobile (video S1 and S2). Some mitochondria observed in epidermal cells and/or in root hairs turned around the filament axis along which they were moving (Fig. 6). Moreover, the mitochondria moved quickly and continuously along the prominent actin tracks, while slower movement with frequent pauses was observed along the fine actin filaments. Most mitochondria moved in a continuous manner in the base of the root hairs, whereas they moved non-uniformly in the subapical and apical regions.

**Figure 7:**
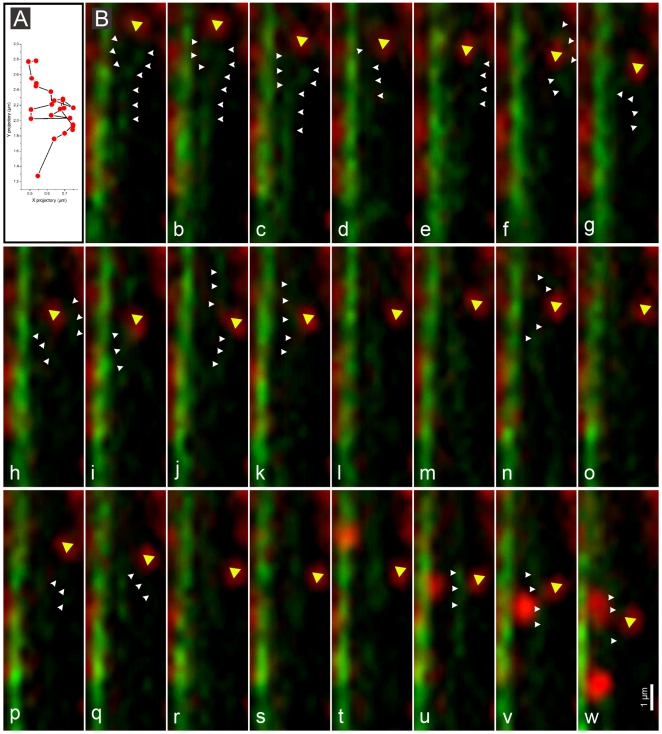
Regulation of mitochondrial movements by actin filament dynamics. A. An x-y plot of mitochondrial movement (yellow arrowhead in images a-w). B. Fluorescence micrographs showing dynamic remodeling of fine actin filaments (white arrowhead) and concomitant changes in mitochondrial movement. When the actin filament was in close proximity to the mitochondrion (yellow arrowhead), the mitochondrion was subject to linear movement (a–c and u–w). When the actin filament underwent rapid remodelling (d–k and n, p, q), the mitochondrion ceased liner movement and underwent a wiggling motion. When the actin filament detached from the mitochondrion (i, m, o and r–t), the mitochondrion became immobile. Mitochondria were labeled with MitoTracker (red), actin was visualized using *GFP-FABD2* (green). The single images in B represent a single frame of a “flat merged” projection (each comprised of 14 images in z-axis) of a 4-D time series. The 1 µm scale bar in w applies for images a-w in this figure.

Further detailed analysis demonstrated that acropetal and basipetal movement of mitochondria occurred on distinct actin filaments, as opposed to the shuttling of mitochondria along the same filament in both directions (video S2). Although these actin filaments displayed frequent aggregation and disaggregation, the direction of mitochondrial movement remained unchanged along a given actin filament. In addition, mitochondria displayed frequent detachment and reattachment to the same or different actin filaments, accompanied occasionally by a shift in the direction of mitochondrial movement. Our data suggest that fine actin filament dynamics control mitochondrial movements: (1) when G-actin assembled into actin filaments adjacent to a mitochondrion, this mitochondrion moved in a linear direction; (2) when the actin filament remodeled quickly around a mitochondrion, the mitochondrion was driven to wiggle; (3) when the actin filament became detached from a mitochondrion, this mitochondrion was immobile (Fig. 7 and video S3).

## Discussion

Mitochondrial movements are complex and hypersensitive to various stimuli, such as temperature [38], [39], pH [40], [41], and light [15]. Sensitivity to such physical perturbations means that exposure of live cells to high illumination over extended periods of time, such as occurs during visualization under a standard confocal microscope, is likely to have adverse effects on mitochondrial movements. EWM minimizes photobleaching and phototoxic reactions due to reduced light scattering of evanescent wave excitation as compared to epi-excitation in a conventional confocal laser-scanning microscope [34], [42], [43]. EWM, therefore, causes less damage over long imaging intervals and/or during relatively long capture times. Furthermore, the increased time resolution afforded by EWM can reveal the true dynamism of organelle movements [44], which is particularly important for imaging mitochondrial movement in the cortical cytoplasm adjacent to the plasma membrane. SDCM, on the other hand, allows imaging deeper within specimens and with much greater temporal resolution. By combining these two imaging techniques we have been able to characterize mitochondria as they undergo linear movements, wiggling motion, or when immobile.

de Win et al. found that the behavior of organelles depended on their position within tobacco pollen tubes [45], although the identity of the organelles was mostly unknown. However, this phenomenon was not reported in studies of mitochondria in tobacco culture cells or protoplasts [17], [18]. In this study, detailed analyses of mitochondrial velocities in root hairs have demonstrated that mitochondrial movement is relatively slow in the tip but becomes gradually more rapid and vectorial with increasing distance from the tip in growing root hairs. Interestingly, in fully-grown root hairs this tip-to-base differential in mitochondrial velocity disappeared. It is apparent, therefore, that mitochondrial movement is under some form of spatio-temporal control. Given the corresponding actin cytoskeleton arrangement, we propose that the status of the actin cytoskeleton plays an important role in mitochondrial movement.

Myosin is a molecular motor that slides along actin filaments using energy released from the hydrolysis of ATP. In *Arabidopsis*, some of the class XI myosins were found in association with mitochondria, suggesting their involvement in their movement [46]–[48]. It has been reported that mitochondrial velocity exhibited a more than 3-fold lower velocity in an *Arabidopsis* myosin XI knock-out mutant than that in the wild type [48], [49]. BDM has been shown to act as an inhibitor of plant myosin, although it can have a side effect on the organization of actin filament bundles at high concentrations [50], [51]. In our study, we found that mitochondrial movements were inhibited by BDM in a dose-dependent manner. 10 mM BDM caused a 30% decline in mitochondrial velocity and no visible changes in the actin cytoskeleton, whereas 30 mM BDM induced a 65% decline in mitochondrial velocity and slightly thicker actin filaments. That mitochondrial velocity was similarly reduced in the myosin XI knock-out [48], [49] as by the treatment with BDM in this study suggests that myosin is not the only factor driving mitochondrial movement in root hairs. Furthermore, it was found that mitochondrial linear movement could not be completely inhibited by Jas, but could by a combined treatment with both 30 mM BDM and Jas. Based on these results, we conclude that myosin and actin filament dynamics cooperate in driving mitochondrial movement in root hairs.

Actin cables are composed of bundles of short actin filaments [52] and these short actin filaments undergo continuous turnover in yeast [20]. Qualitatively similar actin dynamics were also reported in plant cells, where single actin filaments were more dynamic than actin filament bundles or cables [21]. Mitochondria can use cytoskeleton-based mechanisms for movement that are distinct from the well-established mechanism for force generation by myosin along cytoskeletal tracks in yeast [19], [53]. For example, actin polymerization itself can generate the force for mitochondrial movement along a cytoskeletal track [54]. Furthermore, mitochondria can also bind to actin cables undergoing retrograde flow and use the forces of retrograde actin cable flow for passive transport toward the tip of the yeast mother cell [27]. In present study, we found that a large number of single actin filaments displayed dynamic behavior in fast-growing *Arabidopsis* root hairs. Our results clearly show that actin filament elongation does not drive mitochondrial movements, because treatment with CD (CD binds to the barbed end and inhibits filament elongation directly) did not affect mitochondrial velocity until the mitochondria stopped abruptly, or started wiggling in the apical region where fine actin filaments are not detected. When the G-actin pool was sequestered by treatment with LatB, mitochondria lost their velocities although the thick actin filaments remained, suggesting an available G-actin pool is essential for mitochondrial movements. In addition, Jas treatment induced a 42.4% reduction in mitochondrial average velocity in the cortical cytoplasm. Given the effects of Jas on actin turnover [29], [36], [37], these results lead us to conclude that mitochondrial movement can be driven by actin turnover. Based on the findings that actin filaments can slide along other filaments in *Arabidopsis* [21] and that active actin filament flow also occurs in growing lily pollen tubes [22], the most probable explanation for our observations is that mitochondrial movements can be powered by the force of actin flow driven by actin turnover. Moreover, the actin cytoskeleton has been implicated to play key roles in the positioning of mitochondria in plant cells [55]. In animal cells, it was reported that mitochondrial distribution was regulated by RhoA and formins, which function in actin filament organization [56]. In our study it was found, at various developmental stages, that mitochondrial aggregation was correlated with the site where fine actin filaments were arranged. When fine actin filaments disappeared upon treatment of CD and Jas, mitochondria underwent a wiggling motion, or movement was arrested. In addition, simultaneous visualization of mitochondria and the actin cytoskeleton revealed that the transition from vectorial movement of mitochondria to relative immobility is coincident with the disappearance of mitochondrial-associated fine actin filaments. Together, our results suggest that actin filament depolymerization was required for the arrest of mitochondrial movement.

In yeast microtubules are involved in directing the proper placement of actin polymerization through transporting the formin regulators tea1p and tea4p via association with tea2p (kinesin) and tip1p (CLIP-170) [57]. The *Arabidopsis* genome contains homologs of these yeast proteins [58], suggesting conservation of the role of microtubules in controlling actin filament dynamics. Oryzalin is a dinitroaniline herbicide that binds rapidly and reversibly to cytoplasmic tubulin heterodimers, and thereby prevents further growth of microtubules, leading to microtubule depolymerization, starting with the most dynamic microtubules [59], [60], whereas taxol is a drug that stabilize tubulin against depolymerization, thereby inducing the stabilization of microtubules [61], [62]. We found that treatment with oryzalin caused the dispersal and curving of fine actin filaments, whereas treatment with taxol induced actin filament assembly in the apex region, confirming a role for microtubules in the positioning and/or organization of actin filaments. Concomitant with these alterations in actin filament organization, our measurements show that mitochondria moved at more variable instantaneous velocities and with a curved trajectory with more frequent pauses when treated with oryzalin. In contrast, mitochondria moved faster with fewer pauses when treated with taxol. These results show that microtubules play roles in the regulation of mitochondrial velocity and trajectory, rather than simply their position in the cytoplasm as suggested by experiments using cultured cells of tobacco [17]. Given the link between microtubule plus-ends and formin [57], [58], and the link between actin filament organization and formin [63], we speculate that alterations in microtubule dynamics lead directly to a redistribution of formin proteins, which in turn regulates mitochondrial velocity and positioning via variations in actin filament dynamics.

In summary, using a dual labeling technique, together with EWM and SDCM, we have collected data that enable three novel conclusions to be made: (1) myosin and actin turnover cooperate in driving mitochondrial linear movements; (2) the transition of mitochondria from immobility to movement involves *de novo* actin polymerization and depolymerization; (3) microtubule dynamics has profound effects on mitochondrial velocity, trajectory and positioning via its role in directing the arrangement of actin filaments. Taken together, our data can be combined to generate a testable model that expands our insights into the molecular control of mitochondrial movement in plant cells.

## Materials and Methods

### Plasmid constructs

Generation of stably transformed *Arabidopsis* plants expressing GFP-FABD2 is described in Voigt et al. [33] and mitochondria-targeted GFP (mito-GFP) in Logan and Leaver [35]. T3 and T4 generation plants were used in this study.

### Plant culture

According to Valvekens et al. [64], seeds were surface sterilized for 2 min in 70% EtOH, transferred to 5% (w/v) NaClO for 15 min then washed five times with sterilized distilled water. Subsequently, a thin layer of solid medium containing one-half strength Murashige and Skoog salts with 0.7% plant agar (Duchefa, Haarlem, the Netherlands), covered by a 30×24 mm piece of biofoil (Vivascience via Merck, Poole, UK) was placed on a coverslip (50×24 mm). The solid medium that was not covered by biofoil was removed by making a straight cut with a sharp knife. Seeds were placed against the cut agar surface and allowed to germinate into the solid medium. The slides with seedlings were placed with the seeds towards the upper side on 1 mL pipette tips contained within 70 mm Petri dishes, and sealed with parafilm. Plants were cultured at 22°C in a long daylight regime (16 h light, 8 h dark) for 4 days.

### Dye loading

Mitochondria were stained with 200 nM MitoTracker Red CMXRos (Molecular Probes, Eugene, OR, USA) by direct addition of diluted solutions to the culture medium. After 5 minutes incubation with MitoTracker Red, the medium containing the dye was washed three times with fresh liquid medium containing one-half strength Murashige and Skoog salts

### Inhibitor treatments

All chemicals were purchased from Sigma (St Louis, MO, USA) unless otherwise indicated. Stock concentrations of 1 mM taxol, 10 µM LatB and 100 µM Jas (Molecular Probes, Eugene, OR, USA) were made up in DMSO, BDM was prepared fresh for each experiment as 500 mM stock solution in distilled H_2_O. Oryzalin and CD were prepared as 20 mM and 2 mM stocks respectively in 100% ethanol. Appropriate amounts of stock solutions were added to the samples, after labeling with MitoTracker at 25°C. To reduce the possibility of nonspecific effects of the drug treatments, inhibitors were used at low concentrations and applied for a short period of time (5–10 min).

### Spinning disc confocal microscopy (SDCM)

Simultaneous visualization of mitochondria and actin filaments was performed on a spinning-disk confocal microscopy system consisting of a CSU10 spinning disk unit (Yokogawa Electric corporation) and an iXon EMCCD camera device (Andor, Belfast, Northern Ireland), mounted on a IX71 inverted microscope (Olympus, Japan). GFP-FABD2 and MitoTracker Red CMXRos were excited using the 488 and 559 nm lines of an argon ion laser. The objective was a 40×, 1.30 NA Olympus UPLFLN oil immersion lens. Image acquisition was conducted at a lateral resolution of 512×512 pixels and a scan rate of 2 µs/pixel using Andor iQ (Andor, Belfast, Northern Ireland) acquisition software. The exposure was set to minimize oversaturated pixels in the final images. Fluorescence emission with wavelengths of 500–545 nm and 570–670 nm were collected simultaneously through two channels. The collected images were processed using Adobe Photoshop 7.0 (Adobe Systems) and Image J 1.34e (Wayne Rasband, National Institutes of Health, Bethesda, MD, USA).

### Evanescent wave microscopy (EWM)

Variable-angle total internal reflection fluorescence microscopy, also known as EWM as described in a previous paper [34], was used to image mitochondrial movement in *Arabidopsis* root hairs, particularly in the cortical cytoplasm [44]. MitoTracker-labeled mitochondria in root hairs were visualized with an excitation wavelength of 514 nm and an emission wavelength of 575 nm. Fluorescence was gathered through a 100× Apo OHR objective (NA 1.65; Olympus Corp.) at the optimal angle of the incident light. Time-lapse images were acquired every 50 and 200 ms and sampled through a frame grabber with genuine 16 Bit (2^16^, 65536 gray levels). The images were analyzed with Image-Pro Plus 5.1 (Media Cybernetics, Inc., San Diego, CA, USA), Adobe Photoshop 7.0 (Adobe Systems), and Image J 1.34e. The pixel size was 74 nm with a 1.6× optical zoom, and the image size was typically 326×484 pixels. The fluorescence intensity was expressed in the 8-bit value of digitization as previously described [65], [66].

The image stacks were subsequently subjected to analysis using Image J software, which can track the movement of individual mitochondria by ascribing an exact location to the organelle [(x_1_, y_1_), (x_2_, y_2_), (x_3_, y_3_)…(x_n_, y_n_)]. Therefore, the distance (D_n_) of an individual mitochondrial movement during the time of interval (T_n_) can be estimated by the formula below: 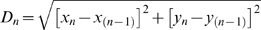



This allows the instantaneous velocity (V_n_) to be determined by:

V_n_ = D_n_/T_n_

## Supporting Information

Figure S1 Dose-dependent effects of BDM on actin filaments organization in growing root hairs visualized using GFP-FABD2. The single pictures represent a stack of all optical sections through these cells visualized under spinning disc confocal microscopy. A.An untreated growing root hair in standard medium. Scale bar = 10 µm. B and C. Growing root hairs treated with 10 and 20 mM BDM for 10 minutes, respectively, showing little change in actin filament organization in comparison with that in untreated growing root hairs. Scale bar = 10 µm. D. A growing root hair treated with 30 mM BDM for 10 minutes. Note slight change in actin filament organization. Scale bar = 10 µm. E, F and G. Growing root hairs treated with 40, 50 and 70 mM BDM for 10 minutes, respectively. Note severe change in actin filament organization. Scale bar = 10 µm.
(10.69 MB TIF)

Figure S2 The effect of BDM treatments on mitochondrial movements in root hairs. The BDM concentrations were set at 0, 10, 20, 30, 40, 50 and 70 mM; The results are from four replicates consisting of approximately 250 mitochondria each.
(2.14 MB TIF)

Video S1 Video sequence showing active mitochondrial movements in an untreated Arabidopsis root hair. Images were collected at 200 millisecond intervals using variable-angle evanescent wave microscope and played back at 10 frames per second (fps). Total elapsed time is 19.2 s.
(7.41 MB AVI)

Video S2 Video sequence showing mitochondrial movements along dynamic actin filaments in an untreated Arabidopsis root hair. Images were collected at 1317 millisecond intervals using spinning disc confocal microscope and played back at 10 fps. Total elapsed time is 52.68 s.
(9.80 MB AVI)

Video S3 Video sequence showing mitochondrial movements controlled by actin filament dynamics. Images were collected at 3.5 second intervals using the spinning disc confocal microscope and played back at 5 fps. Total elapsed time is 112 s.
(8.00 MB AVI)
