# Supplementary figures and images for: Actin Turnover Is Required for Myosin-Dependent Mitochondrial Movements in Arabidopsis Root Hairs
Source: PLoS One. 2009 Jun 18;4(6):e5961. doi: 10.1371/journal.pone.0005961 (PMC2694364; doi:10.1371/journal.pone.0005961)

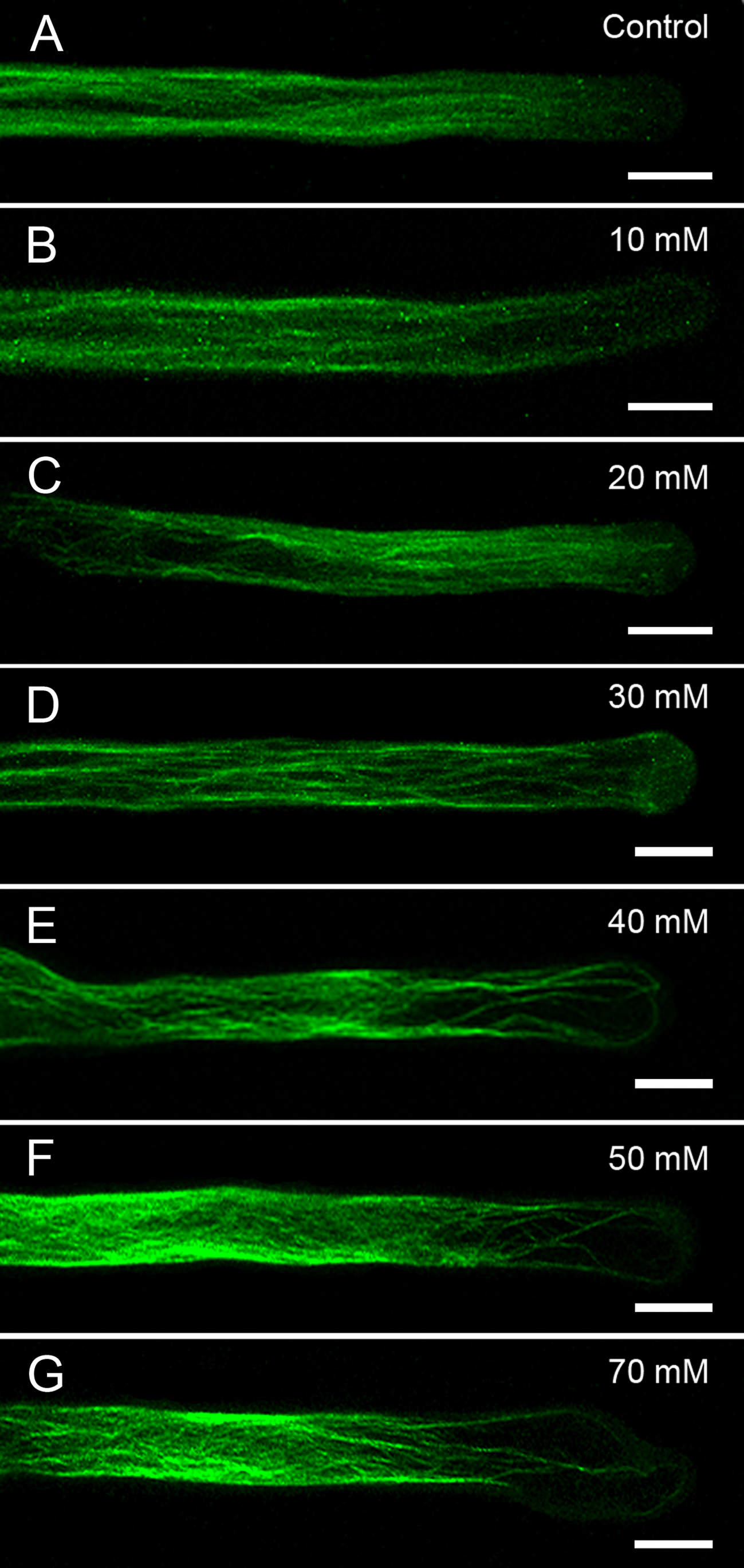

Supplement: Figure S1 — Dose-dependent effects of BDM on actin filaments organization in growing root hairs visualized using GFP-FABD2. The single pictures represent a stack of all optical sections through these cells visualized under spinning disc confocal microscopy. A.An untreated growing root hair in standard medium. Scale bar = 10 µm. B and C. Growing root hairs treated with 10 and 20 mM BDM for 10 minutes, respectively, showing little change in actin filament organization in comparison with that in untreated growing root hairs. Scale bar = 10 µm. D. A growing root hair treated with 30 mM BDM for 10 minutes. Note slight change in actin filament organization. Scale bar = 10 µm. E, F and G. Growing root hairs treated with 40, 50 and 70 mM BDM for 10 minutes, respectively. Note severe change in actin filament organization. Scale bar = 10 µm. (10.69 MB TIF) [file pone.0005961.s001.tif]
